# Exploring How Personality Affects Privacy Control Behavior on Social Networking Sites

**DOI:** 10.3389/fpsyg.2019.01771

**Published:** 2019-07-31

**Authors:** Yuhui Li, Zhaoxing Huang, Yenchun Jim Wu, Zhiqiang Wang

**Affiliations:** ^1^School of Innovation and Entrepreneurship, Wenzhou Medical University, Zhejiang, China; ^2^Graduate Institute of Global Business and Strategy, National Taiwan Normal University and National Taipei University of Education, Taipei, Taiwan; ^3^College of Innovation and Entrepreneurship, Wenzhou University, Zhejiang, China

**Keywords:** privacy, social network sites, personality, personality and behavior, social media

## Abstract

Few studies have examined the relationship between personality traits and social networking sites (SNSs) with a dominant concentration on the personality alterations under SNSs influence. The relationship between personality and privacy control was less focused and discussed. In order to figure out the internal mechanism of such link among youth SNSs users, the Theory of Planned Behavior (TPB) was extended by including Five-Factor Model of Personality to explore how personality traits interact with privacy control behavior on SNSs. The investigation using the theoretical method mentioned led to several hypotheses which were later assessed by an online study conducted within randomly chosen college students (*N* = 201) from two randomly chosen universities in China. This sampling strategy was designed to mimic the situation of targeted research population in the most reasonable way. The results suggested neuroticism and openness predicted SNSs privacy. Neuroticism and openness predicted “networked privacy” was also found. Theoretical implications of these findings were addressed.

## Introduction

Over the past decade, communication through social networking sites (SNSs) has been adopted by young generations all over the world (Boyd, 2007). Through SNSs, people share large pieces of private information. However, such sharing arises a question mark: online safety. SNSs users are extremely vulnerable to identity theft, stalking, insult and blackmail when they upload pictures with relevant feelings and current location. As online risks prevail, youth users tend to set weak instead of zero disclosure for the private information, which is in accordance with the benefit of their social capital. It is reflected that privacy control has been regarded as a vital issue for the prevention of online risks, but active privacy behavior is still an effective way to manage social relationships online (Boyd, 2014).

A considerable number of researches has examined the link between SNS and personality (Marwick and Boyd, 2014; Li et al., 2015; Ohk, 2016; Moon and Um, 2018), however, such work majorly focused on the effects of personality on SNSs usage and self-disclosure (Ross et al., 2009; Winter et al., 2014). The question that has not yet been addressed in this field is the relationship between personality and privacy. Current study attempts to fill this blank.

### Privacy Control on Social Networking Sites

Information control refers to the restriction level when SNSs users halt the flow of social information during interaction when they communicate online (Matshaba, 2018). There are two ways where individuals can block information on SNSs. One is to limit the appearance of certain information. Hypothetically, if a piece of information were inappropriate to be disclosed, users would simply refuse to share it. The other one is to control the access to the information, namely privacy controls. Most SNSs has provided hierarchical privacy settings for their users, which enabled various controls over self-disclosed information and it will only be seen by the selected audience through lifting or lowering access. Our conception of privacy speaks to the behavior to control to whom and to what extent information disclosed on SNSs is communicated to others (Ellison et al., 2011).

Young and Quan-Haase (2013) suggested that SNSs users were not naive. They took a variety of strategies to protect their personal information online. According to our pilot interview, Chinese college students mainly employed three strategies to control what they have disclosed on SNSs: alteration of the default privacy setting, deletion of content from one’s profile and creation of sub-set friend lists to establish varying levels of privacy. The simplest strategy is to alter the default privacy setting. Whereas, users engaging in this strategy may limit their network size and potential online influence. Skilled users of SNSs may conduct more active strategies. For instance, they are highly likely to distribute content to a pre-set group containing certain friends—while keeping these disclosures hidden from the rest. A research (Ellison et al., 2011) examined this strategy then found that users employing segmented privacy settings had larger Facebook networks and higher perceived bridging and bonding social capital than those who do not use this feature. The other research (Stutzman et al., 2008) defined this complicated strategy as networked privacy. It is not a basic denying information. On the contrary, it requires meaningful control over the networked contexts where information flows. Because of its importance and implications for practice, it is necessary to check its association with personality traits individually.

### Theory of Planned Behavior

Theory of Planned Behavior (TPB) was proposed by Ajzen (1985) then strengthened by including perceived behavioral control (PBC) in 1991 (Ajzen, 1985, 1991). TPB is based on the assumption that people usually behave in a sensible manner (Ajzen, 2005). As a framework for interpreting risky behavior, it has been successfully applied to explain SNS behavior including privacy behavior (Saeri et al., 2014; Taneja et al., 2014). Subjective norms, attitude, PBC and intention are the core conceptual variables proposed by TPB (Ajzen, 1985, 1991). The theory postulates that behavior is driven by intention, which is a function of the individual attitude toward the behavior, the subjective norms surrounding the performance of the behavior and the individual perception of the ease or the difficulty with which the behavior can be performed. In terms of the privacy on SNSs, “Attitude” is the individual evaluation of performing the privacy control behavior. “Subjective norm” can be defined as an individual perception of whether or not the privacy control should be performed judged by those who are important to the individual. PBC is an individual’s perception of the ease or the difficulty in controlling privacy on SNSs. Privacy settings can be complicated and confusing, they even change a lot (Stutzman et al., 2013). Many users are unfamiliar with the SNSs privacy settings and frustrated at the misunderstanding of the change (Livingstone, 2008). Facing such a problem, many may leave the privacy setting aside. In addition, SNSs privacy is subjective to the norms and practices of their peer group. Out of these analyses, it is reasonable to hypothesize that:

**H1:** Attitude, subjective norm and PBC indirectly predicts privacy behavior via intention. As these factors change privacy behavioral patterns merely by influencing intentions.
**H2:** Perceived behavioral control can predict privacy behavior directly.

### Five-Factor Model (FFM) of Personality and Privacy Control on Social Networking Sites

Personality traits are closely connected with SNSs usage behavior like status updates and self-disclosure (Ong et al., 2011; Ryan and Xenos, 2011; Moore and McElroy, 2012; Fullwood et al., 2014; Lee et al., 2014). It is a crucial factor in understanding why people behave such way on SNSs. As the classification model of personality traits which has been the most frequently cited, five-factor model (FFM) of personality has been repeatedly found to be linked with SNSs behavior (Ross et al., 2009; Amichai-Hamburger and Vinitzky, 2010; Gosling et al., 2011; Ryan and Xenos, 2011; Moore and McElroy, 2012; Seidman, 2012). Although the previous studies had consistently suggested the connection between SNS behavior and personality, none of them had examined whether personality traits can influence privacy behavior on SNSs. In this paper, it was proposed that FFM may directly or indirectly influence privacy behavior on SNSs.

Five-factor model is composed of five personality factors (Costa and McCrae, 1992; McCrae, 1992; McCrae and John, 1992). (1) *Extroversion*: extraversion is characterized by a tendency to be self-confident, outgoing, active and social; (2) *Agreeableness*: agreeableness is elaborated through altruism and caring, aided by friendliness, attention to others, unselfishness and compliance characterize agreeableness; (3) *Neuroticism*: neuroticism refers to the tendency to experience unpleasant emotions, such as anxiety and depression; (4) *Conscientiousness*: conscientiousness is reflected in discipline, responsibility and orderliness; (5) *Openness to experience*: persons higher in openness are more accepting of change, trying new methods of communication and seeking out new and novel experiences.

Extroverts are sociable and talkative, they tend to use SNSs to present themselves and communicate with friends they meet offline (Gosling et al., 2011; Ong et al., 2011; Lee et al., 2014). By focusing on the positive aspects of situations, extroverts may perceive online risks as less stressful. Since the positivity and energy of extroverts are likely to result in less strain and fewer time pressures, it is expected that extroversion is negatively related to privacy control attitude, intention and behavior. Hereby, the third hypothesis was presented as:

**H3:** Extroversion is negatively associated with privacy behavior on SNSs.

Individuals with higher level on neuroticism were found to be more likely to post private information (Amichai-Hamburger and Vinitzky, 2010; Correa et al., 2010). On the other hand, they tend to be more anxious, tense and insecure. Such characteristics may lead those who self-disclose private information on SNSs (Moore and McElroy, 2012) to regret, which further reinforce their privacy control on SNSs. Thus, the next assumption should be described as following:

**H4:** Neuroticism is positively associated with privacy behavior on SNSs.

Willingness to receive experience is featured by curiosity, open-mindedness and the interest in exploring new ideas. This personality trait is most likely to be associated with privacy setting. Individuals high in openness engaged in increased online sociability through SNSs (Ross et al., 2009; Correa et al., 2010) may tend to have non-private profiles as strict privacy settings may harm their ability to communicate with friends online. Then the fifth hypothesis can be shown as:

**H5:** Openness is negatively associated with privacy behavior on SNSs.

People with characteristics associated with agreeableness may bring them more social supports from friends. They also tend to take advantage of SNSs to obtain more: a greater number of postings, a stronger level of regret about inappropriate contents they may have posted on SNSs (Moore and McElroy, 2012). As such, agreeableness should have positively related with privacy control behavior, which functioned as the last hypothesis:

**H6:** Agreeableness is positively associated with privacy behavior on SNSs.

Certain researches have been conducted on the relationship between conscientiousness and SNSs use. The results summarized that conscientious individuals upload significantly fewer pictures on SNSs and they hardly have anything to protect online. No hypothesis will be applied.

### Current Studies

The overarching goal of current studies is to explore how personality affects privacy behavior on SNSs. First of all, the direct link between FFM and privacy behavior was explored. Then, guided by TPB, the unknown association between personality traits and TPB variables was targeted to examine which traits could have indirect influences on privacy. By integrating both the direct and indirect association, it was able to set up a full model to picture the link between FFM and privacy behavior on SNSs. To finalize the aim, the possible associations between FFM and each specific privacy behavior were also to be discovered.

## Materials and Methods

To evaluate the situation of privacy control behavior for Chinese college students on SNSs, a sample of 231 undergraduates from two universities in Beijing and Tianjin was recruited randomly in 2018. These two universities were also randomly chosen to avoid unscientific factors as any manual division intervened sampling strategies such as stratification or clustering could introduce biased results, considering enormous and complexed differences of culturally, geographically and socio-environmentally influenced habitants among Chinese. An email list of these undergraduates was obtained from the university student office. An invitation with an exclusive QR code was sent to these students, outlining the brief purpose of the study and an incentive of CNY50 which could be earned once they extract the QR code to enter the survey then validly complete the task. The survey was operated in form of online questionnaire where the data could be accumulated and statistically calculated directly. The questions were elaborately generated, combining the coverage of TPB core variables and the convenience of understanding and answering for the interviewees. Summarized from a statistic report, a total of 201 questionnaires were collected out of the 231 in the sample (87%), as it indicated that they had individual accounts on “Renren” (a Chinese SNS). In total, 193 valid questionnaires were collected and 8 were given up because of their missing some important information. Their age ranged from 17 to 23, with an average of 20.27. They were all full-time students, whose income can be excluded as an external factor. About thirty percent of the sample are male (59 persons out of 193) and seventy percent are female (134 persons out of 193).

Similar to Facebook, Renren has functions of news feeding, friending, communicating, etc. The privacy settings in Renren equip users with certain flexibilities such as blacklist: Renren offers a privacy setting shortcut for the users to decide who have access to their personal profiles. Meanwhile, they can also set different access levels for different contents, for instance, basic information, personal records, educational background, career, and so on.

## Measures

Three types of variables, which belong to privacy control behavior, TPB, and FFM, respectively, were treated as measures. These variables were constructed based on the fundamentals in the mainstream field of interest to ensure validity, attempting to offer a panorama for the study. To examine the correlation of variables among privacy control behavior, FFM, and TPB, two path analyses were performed. Path analysis is an extension of multiple regression analysis that allows exploration of hypothesized direct and indirect relationships among variables. The construction of the path models follows a two-step approach. In the first step, the TPB model, which includes **H1** and **H2**, was fitted as output presented in Table A1. In the second step, the modified TPB model was expended by adding FFM variables to screen the one that can significantly predict privacy behavior or to prove that TPB variables have already been covered in the previous model. To supplement the main findings of the study, each specific privacy control behavior was regressed on FFM personality traits to provide more insights for the study. Demographic variables such as gender were entered in the first block; FFM factors were entered in the second block.

### Variables in Privacy Information Control Behavior

Three items were presented on a 5-point Likert scale (1 = totally disagree, 5 = totally agree) to indicate the extent of privacy information control behavior on SNSs (“I have employed a variety of strategies through the SNSs privacy settings to ensure my information should only be seen by those I targeted,” “I have deleted information posted on my SNSs profile” and “I have increased the default privacy settings on SNSs to limit persons who can see my profile”). Each item was developed to measure one basic type of privacy control behavior.

### Variables in Theory of Planned Behavior

The attitude toward privacy information control was inquired by two items. Items answered on a 5-point Likert scale were utilized to measure the extent of the attitude regarding strict privacy settings. Items were averaged to form a scale score. The correlation was 0.55. The degree of tendency that strict level of privacy settings was adopted by a participant under social pressure was inquired by two other items with a correlation of 0.68. PBC was also measured by two items. On a 5-point Likert scale, participants indicated how strongly they disagreed or agreed that they have the knowledge and ability to protect their private information on SNSs and could or not skillfully control who sees their information. Item scores were averaged; higher scores indicated greater perceived control. The correlation between the two items was significant with a number of 0.36. At the end, two 5-point items were developed to measure the respondents’ intention to increase their privacy setting (“I intend to set high level of strict private setting for my SNSs profile”).

### Variables in Five-Factor Model of Personality

Five-factor model, also known as “The Big Five” or “Big Five Personality Inventory” (John et al., 1991), is a 44-item measure. Each item is evaluated on a 5-point Likert scale, ranging from “Strongly Disagree” to “Strongly Agree.” The FFM has shown satisfactory reliability and validity (Srivastava et al., 2003). The internal consistency coefficient for each subscale is convincing: extroversion (α = 0.79), agreeableness (α = 0.72), conscientiousness (α = 0.81), neuroticism (α = 0.79) and openness to experience (α = 0.70).

## Results

**FIGURE 1:**
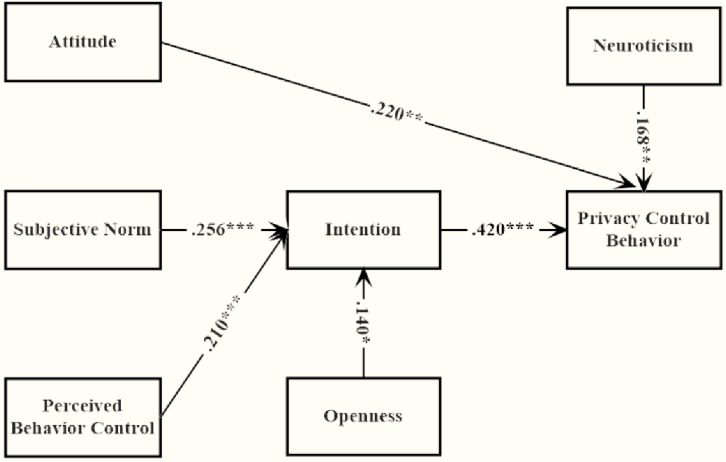
Path coefficient of modified TPB model with MI and EPC. *^∗^p < 0.05, ^∗∗^p < 0.01, ^∗∗∗^p < 0.001*.

All the data collected was processed by software SPSS (Statistical Product and Service Solutions) developed by IBM, which was a prevalent data analysis method within the field of social science. Pearson correlations among measures indicated that all TPB variables were closely associated with each other. Pairwise correlations among attitude, subjective norm, PBC and behavior intention were all significant, confirming the possibility of the proposed TPB structure. Only neuroticism was significantly correlated with privacy behavior as shown in Figure 1, which represented that **H3**, **H5,** and **H6** were disproved while **H4** was corroborated.

### The Original and Modified Theory of Planned Behavior Models

The original TPB model provided an atrocious fit. All of the paths were significant except the one from PBC to privacy information control. Thus, **H1** was supported but **H2** was not. To improve the model fit, the original paths were modified using the raw modification index value (M.I.) and the unstandardized expected parameter change (E.P.C.). The modified TPB model provided a fairly satisfying fit as shown in Table 1. The amount of behavior variance accounted by the TPB variable were 25.7%; the amount of intention variance accounted by the TPB variable were 13.8%. Within this model, one direct path from attitude to behavior was added, see Figure 1. None of the M.I. index exceeded 4.

**TABLE 1:** Fit situation of the original and modified TPB models.

**Parameter**	**Original**	**Modified**
χ^2^(2)	12.122	0.524
*p*_1_	0.002	0.770
*CFI*	0.873	1.000
*TLI*	0.555	1.065
*RMSEA*	0.158	0.000
90%*C.I.*	0.081∼0.248	0.000∼0.093
*p*_2_	0.013	0.852
*SRMR*	0.052	0.009
*AIC*	973.162	961.121
*BIC*	1002.981	990.940

### The Extended Theory of Planned Behavior Model With Five-Factor Model Variables

It was explored whether paths from FFM to privacy behavior and intention were significant. As a result, only the path from openness to intention and the path from neuroticism to privacy behavior were significant, see Figure 1. The extended TPB model provided a perfect fit that was shown in Table 2. Compared with values of the original TPB model, the AIC and BIC values were lower, indicating this extended model was better than the original model. The extra behavior variance accounted by the personality variable were 3.17%. None of the M.I. index exceeded 4.

**TABLE 2:** Fit situation of the original and extended TPB models.

**Parameter**	**Original**	**Extended**
χ^2^(2)	12.122	4.231
*p*_1_	0.002	0.517
*CFI*	0.873	1.000
*TLI*	0.555	1.020
*RMSEA*	0.158	0.000
90%*C.I.*	0.081∼0.248	0.000∼0.091
*p*_2_	0.013	0.745
*SRMR*	0.052	0.025
*AIC*	973.162	935.820
*BIC*	1002.981	968.753

**FIGURE 2:**
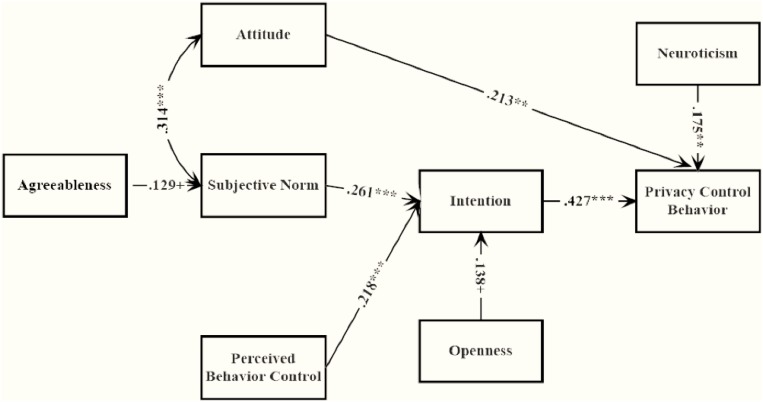
Path coefficient of TPB model with agreeableness. *^∗^p < 0.05, ^∗∗^p < 0.01, ^∗∗∗^p < 0.001*.

It was also explored whether any paths from personality traits to attitude, subjective norms and PBC were significant. The results showed that the mere path from agreeableness to subjective norms was significant, see Figure 2. The model with agreeableness provided an acceptable fit of data: χ^2^(14) = 15.934, *p* = 0.317, *CFI* = 0.982, *TLI* = 0.977, *RMSEA* = 0.027, 90%*C.I.* = 0.000∼0.077, *p* = 0.725, *SRMR* = 0.044. But the *AIC* and *BIC* values were relatively unacceptable as AIC = 1931.034 and BIC = 1983.402, suggesting the model is not as smart as previous one which was extended with FFM.

### The Regression of Specific Privacy Control Behavior on Five-Factor Model

According to Table 3, the effect of openness was significant in predicting “alteration of default privacy setting” and “employ strategies to make information only be seen by targeted audience” when demographic variables were under control. Besides, neuroticism was found to be significantly associated with “employ strategies to make information only be seen by targeted audience.”

**TABLE 3:** Regression analysis on individual privacy behavior.

	Alter privacy setting	Delete information	Targeted audience
Intercept	0.47	0.42	–3.34
**Control variables**			
Age	0.05	0.08	0.16^∗^
Gender	0.27	0.00	–0.04
Family Income	−0.19^∗^	0.26^∗∗^	0.12
*R* ^2^	0.04	0.04	0.03
**Personality**			
Externality	–0.14	–0.25	–0.11
Agreeableness	–0.07	0.11	0.00
Conscientiousness	0.12	–0.24	0.15
Neuroticism	0.11	0.25	0.40^∗^
Openness	0.06^∗∗^	0.01	0.06^∗^
*R* ^2^	0.10	0.11	0.09

**^∗^p < 0.05*, *^∗∗^p < 0.01**

## Discussion

**FIGURE 3:**
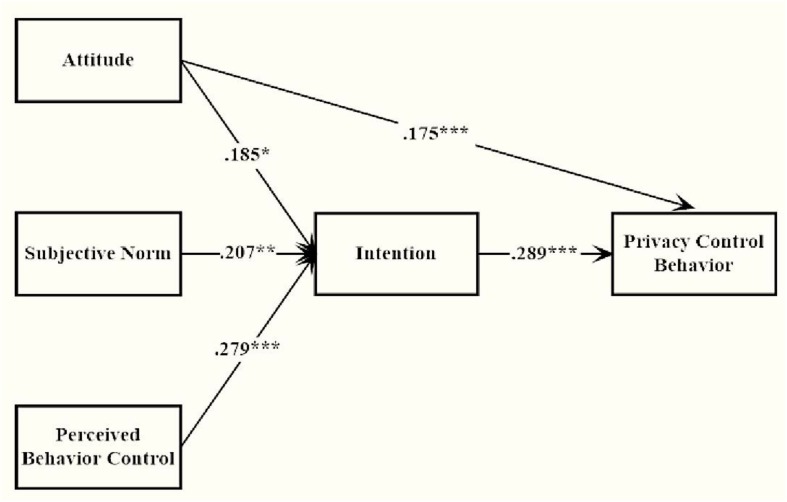
Path coefficient of modified TPB model. *^∗^p < 0.05, ^∗∗^p < 0.01, ^∗∗∗^p < 0.001*.

The results offered considerable confirmation for the proposed purpose, which was lending empirical support to the framework integrating the TPB and personality perspectives. Combining the corroborated **H1** and disproved **H2**, it could be summarized that attitude, subjective norm and PBC were positively associated with behavior only indirectly via intention. At the first level, these variables could be understood as important predictors for privacy behavior, but results also suggested they were important mediators in the relationship between personality and privacy behavior. Although the interest was in how personality traits were associated with privacy behavior, it was still beneficial to notice the relationship of these variables. See Figure 3.

When personality traits were added into the model as described through **H3**, **H4**, **H5,** and **H6**, only **H4** was not rejected by the results. Namely, extroversion, neuroticism and openness were positively correlated with privacy behavior on SNSs while agreeableness was negatively correlated. In accordance with the findings from Butt and Phillips (2008), those with high level of neuroticism were more likely to control what information was shared on SNSs. Among the five traits examined, neuroticism was the most relevant to privacy control behavior on SNSs, see Figure 1. This was consistent with the recent personality studies on SNSs self-disclosure (Amichai-Hamburger and Vinitzky, 2010; Wang et al., 2012). Their findings demonstrated that highly neurotic users conducted more status updates and posted more selfies to identify themselves on SNSs as it was able to give the user plenty of time to consider what he/she wanted to include in his/her SNS profile. Under common circumstances, the more active SNSs users were, the more private they inclined to be on SNSs. Further individual analysis revealed that those with high level of neuroticism were not worried about their self-presentation online. On the contrary, they were masters of these privacy settings, setting varying levels of privacy for different friends and allowing only appropriate contents to be seen by the audience they targeted.

Openness was featured by curiosity, open-mindedness and their willingness to explore new ideas (McCrae and John, 1992). It was reasonable to hypothesize that individuals with high level of openness were engaged in low levels of privacy information control (Ross et al., 2009). On the contrary to this intuitive hypothesis, the results implied that openness was positively and indirectly associated with privacy behavior. The following regression confirmed this positive association and further suggested that individuals with high level of openness not only increased their default privacy setting but also employed different self-presentational strategies for different targeted audience groups. One possible explanation for these findings was that higher level of openness was correlated with a greater tendency toward the social functions of SNSs. They spent more time, had more friends and posted more photos on SNSs (Kuo and Tang, 2014), representing that they had more online risks and had to deal with more collapsed contexts and social norms.

The results also lent support to the idea that those with high level of agreeableness were more likely to take similar private strategies on SNSs to their friends. These findings were sensible in the way that people with an inclination to caring, trusting, sympathetic and cooperative were expected to be more vulnerable to their friends’ behavior. However, it was also important to emphasize that, though agreeableness was relevant to privacy through subjective norms, its effect was minor (β = *0.129, p* = *0.06*). The extroverts uploaded photos and updated status more frequently and displayed more friends on SNSs (Amichai-Hamburger and Vinitzky, 2010; Gosling et al., 2011; Ryan and Xenos, 2011). Following the intuition, those who were active on SNSs were also private, the extrovert was expected to be cautious about what they disclosed on SNSs. Surprisingly, the results were not consistent with this intuition.

It was hardly to achieve true privacy on SNSs, as context boundaries were blurred by the persistent and searchable nature of SNSs. Additionally, posted information like status updates were automatically archived, being accessible at any time. It was complex to conduct privacy behavior on SNSs, requiring attention to manage personal impression across a variety of contexts and relationships. Lifting the default privacy setting level would lead to a great loss of targeted audience on SNSs. On the contrary, navigating audience through SNSs and employing different self-presentational strategies for different groups and individuals on the site may result in more social capital bonus (Ellison et al., 2011). To derive social capital, it was beneficial to confirm whether personalities were associated with the privacy behavior. An evident implication of results for the online privacy literature was that individuals with high level of neuroticism and openness were more likely to employ this strategy. These people could be good at navigating technology and understanding the context in which they operated and influenced others’ behavior, shaped who can interpret what information and possessed the knowledge and skills necessary to directly affected how information flowed and was interpreted within that context (Marwick and Boyd, 2014).

## Conclusion

Expanding previous researches, this study contributed to our understanding of who would protect privacy on SNS and who conducted what kind of privacy behavior on SNSs. Understanding of these questions was extremely vital in several aspects. The findings had implications for SNSs privacy feature design. Digging the different roots without the influence of external factors such as income, people used SNSs privacy settings from TPB perspective and assessed who conduct what kinds of privacy behavior. It could help SNSs developers with better understanding of the diversified needs of their customers. Besides, these findings provided insights into SNSs privacy practices and shed light on who are more likely to negotiate context in social media.

This study improved the understanding of SNSs privacy through discovering a causative link between personality and privacy control behavior. However, under interpreting the results of the study, one must pay attention to the limits. The cross-sectional study design did not allow causal inferences. Accordingly, this study should be interpreted as correlative. Secondly, the proposed model merely focused on the five personality traits within FFM. The personality traits included in our model only explained a small portion of the variance in “privacy behavior on SNSs.” Future research should continue to search for relevant personality antecedents of SNSs privacy behavior to improve the model’s explanatory power. An additional limitation was that the study only focused on privacy behavior and had no measure of self-disclosure on SNS. Self-disclosure, as suggested by Ellison et al. (2011), was the key to extracting relational benefits from the use of SNSs. It complicated privacy’s relationship with personality.

## Data Availability

All datasets generated for this study are included in the manuscript and/or the supplementary files.

## Ethics Statement

Ethics approval for this research was not required as per institutional and national guidelines. Participant consent was obtained by virtue of completing the questionnaire.

## Author Contributions

YL wrote the manuscript, reviewed the literature, and analyzed the data. ZH contributed to the research topic and framework. YW wrote the strategy and edited the manuscript. ZW performed the technical analysis.

## Conflict of Interest Statement

The authors declare that the research was conducted in the absence of any commercial or financial relationships that could be construed as a potential conflict of interest.

## Appendix

**TABLE A1:** Measure correlations.

	**Intention**	**Attitude**	**Norm**	**PBC**	**Externality**	**Agreeable**	**Contentious**	**Neuroticism**	**Openness**
Behavior	0.46^∗∗^	0.33^∗∗^	0.22	0.10	–0.03	–0.06	–0.05	0.18^∗^	0.10
Intention	1.00	0.25^∗∗^	0.24^∗∗^	0.23^∗∗^	–0.02	0.00	0.07	0.00	0.17^∗^
Attitude	−	1.00	0.37^∗∗^	0.02	0.00	0.08	0.03	0.07	0.16^∗^
Norm	−	−	1.00	0.02	0.01	0.18	–0.03	–0.02	0.03
PBC	−	−	−	1.00	0.06	0.10	0.14	–0.11	0.06
Externality	−	−	−	−	1.00	0.28^∗∗^	0.25^∗∗^	–0.45^∗∗^	0.45^∗∗^
Agreeable	−	−	−	−	−	1.00	0.29^∗∗^	–0.49^∗∗^	0.28^∗∗^
Contentious	−	−	−	−	−	−	1.00	–0.31^∗∗^	0.22^∗∗^
Neuroticism	−	−	−	−	−	−	−	1.00	–0.30^∗∗^
Openness	−	−	−	−	−	−	−	−	1.00

**^∗^p < 0.05, ^∗∗^p < 0.01*.*
